# PHYSIOLOGICAL LINKAGE WITH CAREGIVERS IS ASSOCIATED WITH EMOTION RECOGNITION IN CARE RECIPIENTS

**DOI:** 10.1093/geroni/igad104.1836

**Published:** 2023-12-21

**Authors:** Kuan-Hua Chen, James Casey, Casey Brown, Jennifer Merrilees, Robert Levenson

**Affiliations:** University of Nebraska Medical Center, Omaha, Nebraska, United States; University of California, Berkeley, Berkeley, California, United States; Georgetown University, Washington, District of Columbia, United States; University of California, San Francisco, San Francisco, California, United States; University of California, Berkeley, Berkeley, California, United States

## Abstract

Persons with neurodegenerative diseases (PWDs) often develop profound declines in the ability to recognize emotions in others that lead to diminished social connectedness with their loved ones. Physiological linkage, which refers to the degree that people’s physiological responses change in coordinated ways, can serve as a proxy measure for social connectedness. Physiological linkage has been found to be diminished during interactions between PWDs (i.e., care recipients) and their family caregivers. However, the association between diminished physiological linkage and PWDs’ lower emotion recognition has not been determined. In a sample of 28 PWD-family caregiver dyads, we quantified physiological linkage as the positive (“in-phase”) correlations between PWD’s and caregiver’s somatic activity. Somatic activity was measured remotely via actigraphy using wristwatches worn by both partners in their homes during waking hours for 7 days. PWDs’ emotion recognition was quantified as the ability to recognize positive and negative emotions of their caregivers accurately during a 10-minute conflict conversation in our laboratory prior to the 7-day home assessment. Lower emotion recognition by PWDs of their caregivers’ negative emotions (β = .49, p = .01), but not positive emotions (β = .12, p = .52) in the laboratory assessment was associated with lower physiological linkage between PWDs and caregivers in their home. Establishing a relationship between PWDs’ deficits in emotion recognition and diminished physiological linkage during conversations with their caregivers provides a basis for understanding problems in establishing social connections that are often observed in PWDs.

